# Sphingosine Promotes Fiber Early Elongation in Upland Cotton

**DOI:** 10.3390/plants13141993

**Published:** 2024-07-21

**Authors:** Li Wang, Changyin Jin, Wenqing Zhang, Xueting Mei, Hang Yu, Man Wu, Wenfeng Pei, Jianjiang Ma, Bingbing Zhang, Ming Luo, Jiwen Yu

**Affiliations:** 1 Zhengzhou Research Base, State Key Laboratory of Cotton Biology, Zhengzhou University, Zhengzhou 450001, China; wangli07-2@163.com (L.W.); jinchangyin1207@163.com (C.J.); zhangwenqing45@163.com (W.Z.); 18699718321@163.com (X.M.); yuhang4102022@163.com (H.Y.); wuman2004@163.com (M.W.); peiwenfeng1988@163.com (W.P.); mjj1699@126.com (J.M.); 18439247312@163.com (B.Z.); 2 State Key Laboratory of Cotton Biology, Institute of Cotton Research, Chinese Academy of Agricultural Sciences, Anyang 455000, China; 3 Key Laboratory of Biotechnology and Crop Quality Improvement of Ministry of Agriculture, Biotechnology Research Center, Southwest University, Chongqing 400716, China

**Keywords:** cotton, fiber elongation, sphingosine, sphingolipidomics, transcriptomics

## Abstract

Sphingolipids play an important role in cotton fiber development, but the regulatory mechanism is largely unclear. We found that serine palmitoyltransferase (SPT) enzyme inhibitors, myriocin and sphingosine (dihydrosphingosine (DHS) and phytosphingosine (PHS)), affected early fiber elongation in cotton, and we performed a sphingolipidomic and transcriptomic analysis of control and PHS-treated fibers. Myriocin inhibited fiber elongation, while DHS and PHS promoted it in a dose–effect manner. Using liquid chromatography–tandem mass spectrometry (LC–MS/MS), we found that contents of 22 sphingolipids in the PHS-treated fibers for 10 days were changed, of which the contents of 4 sphingolipids increased and 18 sphingolipids decreased. The transcriptome analysis identified 432 differentially expressed genes (238 up-regulated and 194 down-regulated) in the PHS-treated fibers. Among them, the phenylpropanoid biosynthesis pathway is the most significant enrichment. The expression levels of transcription factors such as *MYB*, *ERF*, *LBD*, and *bHLH* in the fibers also changed, and most of MYB and ERF were up-regulated. Auxin-related genes *IAA*, *GH3* and *BIG GRAIN 1* were up-regulated, while *ABPs* were down-regulated, and the contents of 3 auxin metabolites were decreased. Our results provide important sphingolipid metabolites and regulatory pathways that influence fiber elongation.

## 1. Introduction

Cotton is the most important natural fiber crop in the world. Fiber development can be divided into four stages, namely fiber initiation, elongation, secondary wall synthesis, and maturation. The elongation stages of cotton fiber mainly depend on cell polar growth, which determines fiber length; therefore, cotton fiber elongation is closely related to fiber yield and quality. Although several plant hormones and important fiber development genes are proven to play important roles in cotton fiber elongation, the regulatory mechanisms of fiber cell elongation remain poorly understood [1].

Sphingolipids are a class of lipid molecules widely present in eukaryotes and composed of sphingosine (also known as long chain base (LCB)), fatty acids, and polar head groups [2]. Sphingolipids are components of lipid rafts in the structural microregions of cell membrane, which affect the physicochemical properties of biofilms [3]. In mammals, sphingolipids affects cell proliferation, cell death, autophagy, inflammation, multidrug resistance, or cell movement [4]. In plants, sphingolipids are also involved in important pathological and physiological processes, including plant–pathogen interactions, cell signaling, stress responses, and programmed cell death processes [5,6,7,8,9].

Sphingosine is the simplest sphingolipid and a component of other complex sphingolipids. Dihydrosphingosine (d18:0, DHS) is the simplest form of sphingosine, which can be hydroxylated to form phytosphingosine (t18:0, PHS) [2]. d18:0 and t18:0 can be desaturated, phosphorylated, or acylated to produce various forms of sphingosine and ceramide [3]. Sphingosine is synthesized de novo by serine palmitoyltransferase (SPT) enzymes, reduced by 3-ketodihydrosphingosine reductase (KDSR), desaturated by sphingolipid-Δ8 (E/Z) desaturase (SLD), and hydroxylated by sphingoid base hydroxylase (SBH) to form different forms of sphingosine molecules [3]. In Arabidopsis, LCB1 and LCB2 are the subunits of SPT, and the small subunit ssSPT can improve the activity of SPT, the orosomucoid protein (ORM) negatively regulates the activity of SPT by interacting with ssSPT [10]. The male gametophytes of loss-of-function mutants of the *LCB1* gene, *fumonisin B (1) resistant11-2 (fbr11-2*) and *LCB2* (*LCB2a* and *LCB2b*) gene, *lcb2a*/*lcb2b,* are lethal [11,12]. The *orosomucoid protein* (*orm1*/ *orm2*) double mutant had impaired embryo development and excessive ceramide accumulation [13]. Abnormal leaf ion content and root iron homeostasis occurred in the *3-ketodihydrosphingosine reductase* (*ksr-1*) mutants [14]. The *sphingoid base hydroxylase* (*sbh1*/*sbh2*) double mutant is dwarfed, and cell elongation and division are inhibited [15]. Studies have shown that injection of sphingosine (d18:1, d18:0 and t18:0) into Arabidopsis leaves can induce programmed cell death, which can be inhibited by LCB-P [16,17]. The addition of sphingosine to tobacco BY-2 cells induced cytosolic Ca^2+^ transient and ROS generation, resulting in the production of programmed cell death [18]. Thus, these studies show that sphingosine is involved in many biological processes of plant growth and development.

In our previous studies, we found that the sphingolipid synthesis inhibitor, fumonisin B1 (FB1), severely inhibited the elongation of cotton fibers and caused sphingolipids in fibers and embryos to be disordered, with most simple sphingolipids increasing and complex sphingolipids decreasing [19]. Sphingosine is the most abundant sphingolipid molecule in different fiber and embryo development stages, and SPT enzyme inhibitor, myriocin, inhibits fiber and embryo growth [20,21]. However, the function and mechanism of sphingosine in cotton fiber elongation are still unclear. The results of this study show that myriocin inhibited fiber elongation in a dose-dependent manner, and low concentrations of myriocin can significantly reduce fiber length, while DHS and PHS promote early elongation of cotton fibers. Furthermore, sphingolipidomic and transcriptomic analyses were performed on the cotton fibers treated with PHS to identify relevant key sphingolipids and biological pathways in cotton fiber elongation. These studies provide new insights into the mechanism of cotton fiber elongation.

## 2. Results

### 2.1. Sphingosine Promotes Cotton Fiber Early Elongation

**Figure 1:**
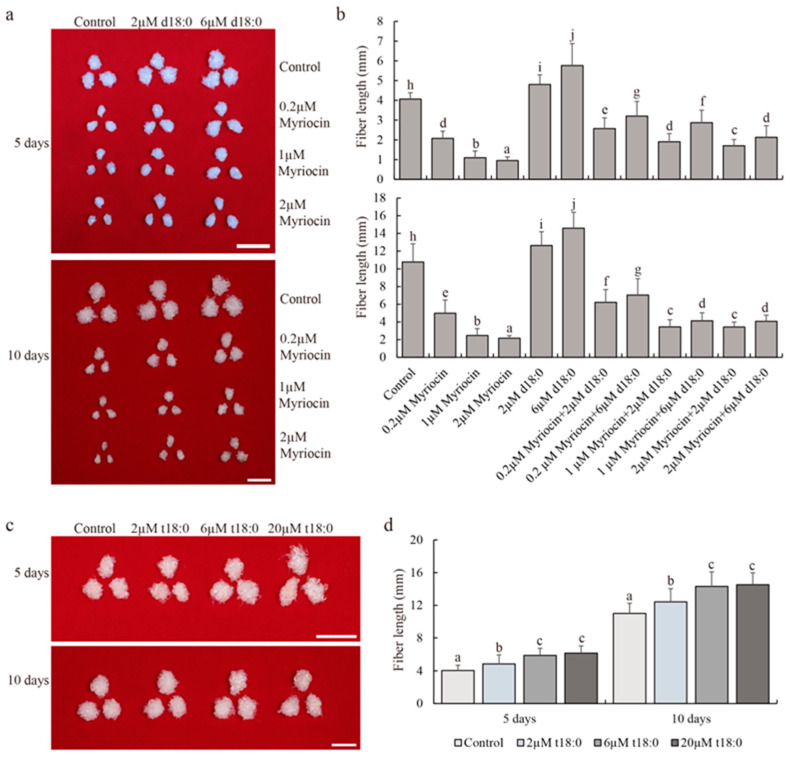
Sphingosine promotes cotton fiber elongation. (**a**) Phenotypic characteristics of myriocin- and DHS-treated fibers after 5 and 10 days of in vitro culture at different concentrations. Scale bars = 1 cm. (**b**) The length of myriocin- and DHS-treated fibers after 5 days (**upper panel**) and 10 days (**lower panel**) at different concentrations. Values represent means ± SD (*n* = 24). (**c**) Phenotype of PHS-treated fibers after 5 and 10 days at different concentrations. Scale bars = 1 cm. (**d**) The length of PHS-treated fibers after 5 and 10 days at different concentrations. Values represent means ± SD (*n* ≥ 24). Different letters above the bars indicate significant differences at the *p* = 0.05 level.

Myriocin is a specific inhibitor of SPT enzyme. We used an in vitro ovule culture system and added three concentrations of myriocin (0.2 µM, 1 µM, and 2 µM) to the culture medium for 5 and 10 days and found that myriocin could significantly inhibit cotton fiber elongation in a dose–effect manner (Figure 1a,b). Myriocin at low concentrations (0.2 µM) significantly inhibited fiber elongation, decreasing fiber length by 49.0% and 53.8% after 5 and 10 days of culture, respectively (Figure 1b). At the same time, DHS, a downstream product of the SPT enzyme, was added for treatment, and it was found that DHS could partially restore the inhibitory effect of myriocin on cotton fiber elongation (Figure 1a,b). The results also indicated that myriocin inhibited the synthesis of sphingosine, resulting in cotton fiber.

Although DHS can promote the elongation of cotton fiber, the content of PHS is the highest in all stages of cotton fiber development, and PHS is the direct product of DHS through hydroxylation. Therefore, we used different concentrations of PHS for ovule culture to observe the development of cotton fiber (Figure 1c). The results showed that the fiber length of PHS-treated with 2 µM, 6 µM, and 20 µM for 5 days increased by 19.5%, 44.7%, and 51.6%, respectively (Figure 1d). The fiber length increased by 12.8%, 29.9%, and 31.9% after 10 days of treatment, respectively (Figure 1d). These results indicated that PHS could promote cotton fiber elongation.

### 2.2. PHS Regulates Sphingolipid Homeostasis in Cotton Fibers

**Figure 2:**
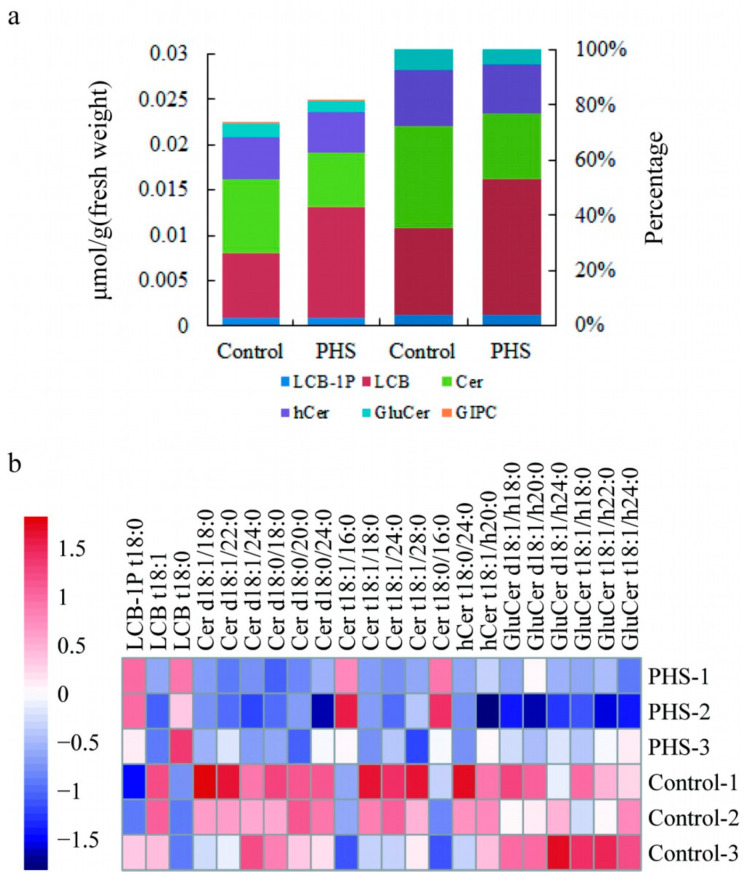
Changes in the sphingolipid content between the control and the PHS fibers (**a**) The total content and percentages of six major sphingolipid categories in the control and the PHS fibers cultured for 10 days. (**b**) Heat map of the individual sphingolipids between the control and the PHS fibers cultured for 10 days.

PHS is a simple sphingolipid; however, the biosynthesis pathway of sphingolipids is complex. It is not clear whether exogenous PHS regulates the content of other sphingolipids in cotton fibers and thus regulates cotton fiber elongation [19]. To analyze which sphingolipids might affect cotton fiber development, we analyzed them by LC–MS/MS. We found that the total content of sphingolipids in PHS-treated fibers was not significantly different from that in the control, but the contents of three categories of sphingolipids changed, among which the content of LCB increased by 71.74%, while the content of Cer and GluCer decreased by 27.34% and 19.88%, respectively (Figure 2a, Table S2). There were no significant differences in the total contents of LCB-1P, hCer, and GIPC of the other three major sphingolipids (Figure 2a, Table S2). In the control, the percentages of LCB, Cer, and GluCer were 31.64%, 36.83%, and 7.42%, respectively (Figure 2a). In PHS-treated fibers, the percentages of LCB, Cer, and GluCer were 48.99%, 24.13%, and 5.36%, respectively (Figure 2a).

This study further analyzed the changes in individual sphingolipid content and found that a total of 22 individual sphingolipids differed in the control and PHS-treated fibers, with 4 sphingolipids increasing and 18 sphingolipids decreasing (Figure 2b). The exogenous application of PHS resulted in the contents of PHS (LCB t18:0) and LCB-1P t18:0 in PHS-treated cotton fibers were 2.23 and 1.33 times higher than those in the control, respectively, while the content of another sphingolipid LCB t18:1 was reduced by 19.94%. These results indicated that PHS treatment could not only absorb more PHS, but also lead to an increase in phosphorylated product and a decrease in desaturated product downstream of LCB t18:0. There were also two Cers containing long-chain fatty acids (16:0-FA), Cer t18:1/16:0 and Cer t18:0/16:0, which were 1.73 and 1.94 times higher than the control samples, respectively. The other 10 types of Cer (d18:1/18:0, d18:1/22:0, d18:1/24:0, d18:0/18:0, d18:0/20:0, d18:0/24:0, t18:1/18:0, t18:1/24:0, t18:1/28:0, t18:0/24:0) and one hCer (t18:1/h20:0) decreased by 20.35%, 27.54%, 45.04%, 43.98%, 38.04%, 34.45%, 38.7%, 30.64%, 29.57%, 29.04%, and 11.02%, respectively. The contents of six GluCer (d18:1/h18:0, 18:1/h20:0, d18:1/h24:0, t18:1/h18:0, t18:1/h22:0, t18:1/h24:0) decreased by 27.79%, 17.36%, 27.33%, 34.51%, 19.47%, and 17.54%, respectively, compared with the control. These differences in sphingolipid molecules between the control and PHS-treated fibers may play an important role in cotton fiber elongation.

### 2.3. Transcriptome Analysis of PHS Treated Fibers

**Figure 3:**
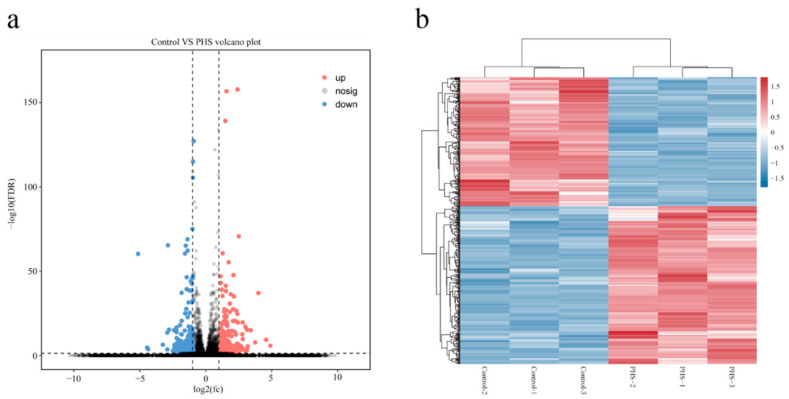
Overview of RNA-SEQ analysis after PHS treatment. (**a**) Volcanic map of differential expression genes in fiber treated with PHS. Blue is the down-regulated gene; red is the up-regulated gene. (**b**) Correlation heat maps of control and PHS-treated fibers.

In order to further explore the molecular mechanism of PHS regulation on cotton fiber growth, six cDNA libraries were constructed from fibers treated with 6µM PHS for 10 days and control for transcriptome analysis. After sequencing the cDNA library, the number of reads in each library ranged from 40,332,094 to 49,055,648, and more than 96.76% of the reads were mapped to the genome of upland cotton. The Q30 of the six libraries was greater than 93.30%, and the average GC content was 44.74% (Table S3). These data indicated that the library-generated reads were suitable for differential gene expression analysis. There were 238 up-regulated genes and 194 down-regulated genes between the control and PHS-treated fibers (Figure 3a,b, Table S4).

**Figure 4:**
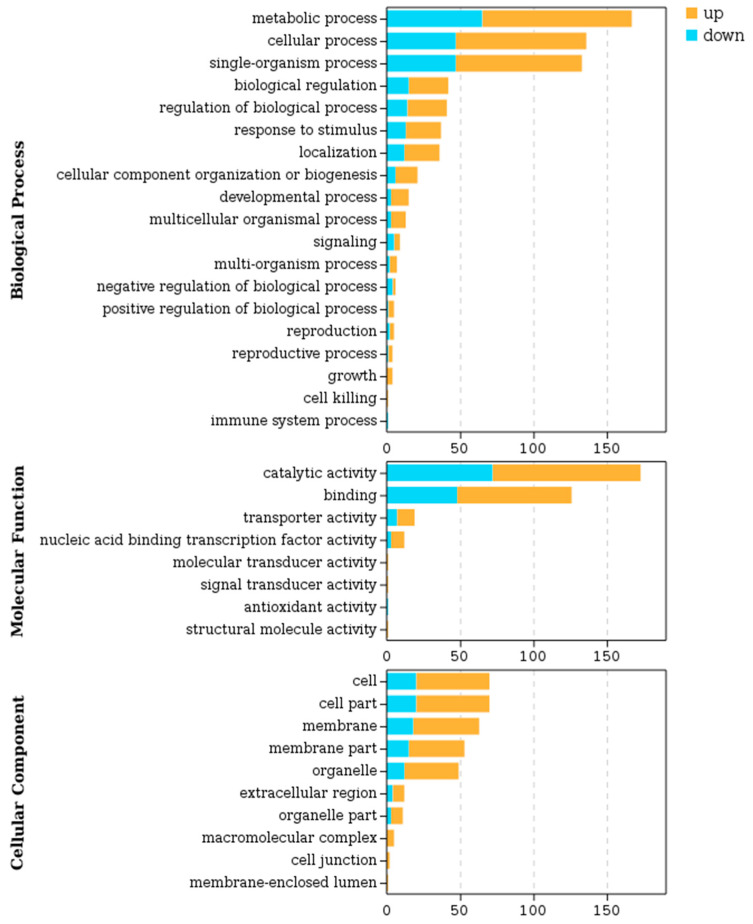
GO enrichment analysis of fibers treated by PHS. Histogram of GO entries with significant enrichment of differentially expressed genes in control and PHS-treated fibers. The height of the column represents the number of genes contained in the pathway, with yellow representing up-regulated genes and blue representing down-regulated genes.

GO enrichment analysis of differential genes was performed using GO database. 116, 227, and 212 differentially expressed genes were assigned to three GO items: cell component, molecular function, and biological process, respectively, which could be divided into 37 functional subcategories (Figure 4). When classifying biological processes, metabolic process, cellular process, and single-organism metabolic process had the highest number of differentially expressed genes. Catalytic activity and binding had the highest number of differential genes in the molecular function classification. In the cell group classification, cell, cell part, membrane, and membrane part had the largest number of differential genes (Figure 4).

**Figure 5:**
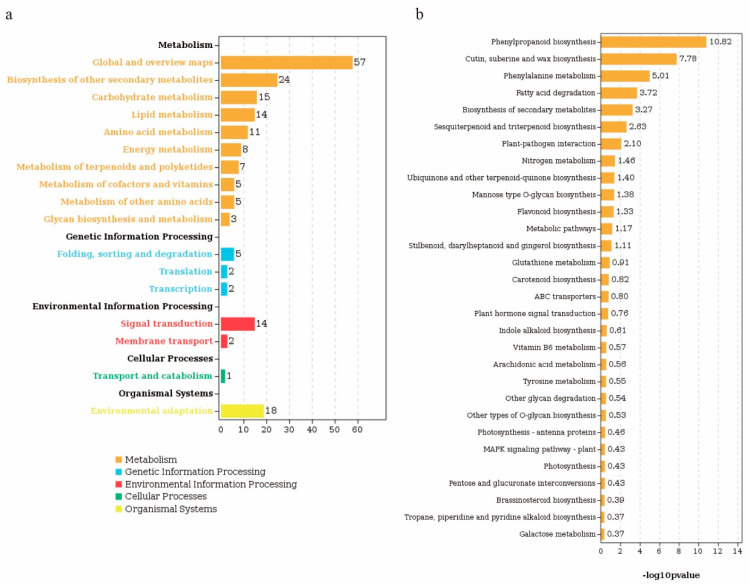
KEGG enrichment analysis of fibers treated with PHS. (**a**) The number of genes annotated into different pathway secondary classifications in different comparison groups. The abscissa is the number of genes, the ordinate is the name of the pathway, each column represents a pathway, and the height of the column represents the number of genes contained in the pathway. Different colors represent different first-level categories. (**b**) Histogram of KEGG enrichment of differentially expressed genes in control and PHS fibers. Each column represents a pathway, and the column height represents the enrichment significance of the pathway, and the value is equal to the −log10 value of the P/Q value of the pathway.

The biological pathways of the differentially expressed genes were identified using a KEGG analysis. A total of 16,134 unigenes and 115 DEGs were divided into 60 known pathways, which were divided into 5 categories, including metabolism, genetic information processing, environmental information processing, cellular processes, and organismal systems, with 10, 3, 2, 1, and 1 sub-categories, respectively (Figure 5a, Table S5). The pathway with the largest number of differentially expressed genes was the metabolic pathway. Most of the differentially expressed genes were involved in the biosynthesis of other secondary metabolites, carbohydrate metabolism, lipid metabolism, amino acid metabolism, and energy metabolism pathways, with 24, 15, 14, 11, and 8 genes, respectively (Figure 5a). The differentially expressed genes in the signal transduction pathway in the environmental-information-processing and the environmental adaptation pathway in the organismal systems were 14 and 18, respectively (Figure 5a). The KEGG pathway analysis of differentially expressed genes showed that the pathways with the highest enrichment were phenylpropanoid biosynthesis (−log10 (*p* = 10.82)), cutin, suberine and wax biosynthesis (−log10 (*p* = 7.78)), phenylalanine metabolism (−log10 (*p* = 5.01)), fatty acid degradation (−log10 (*p* = 3.72)), and biosynthesis of secondary metabolites (−log10 (*p* = 3.27)), sesquiterpenoid and triterpenoid biosynthesis (−log10 (*p* = 2.63)), and plant–pathogen interactions (−log10 (*p* = 2.10)) (Figure 5b).

### 2.4. Sphingosine Regulates Phenylpropanoid Biosynthesis Pathway in Cotton Fibers

**Figure 6:**
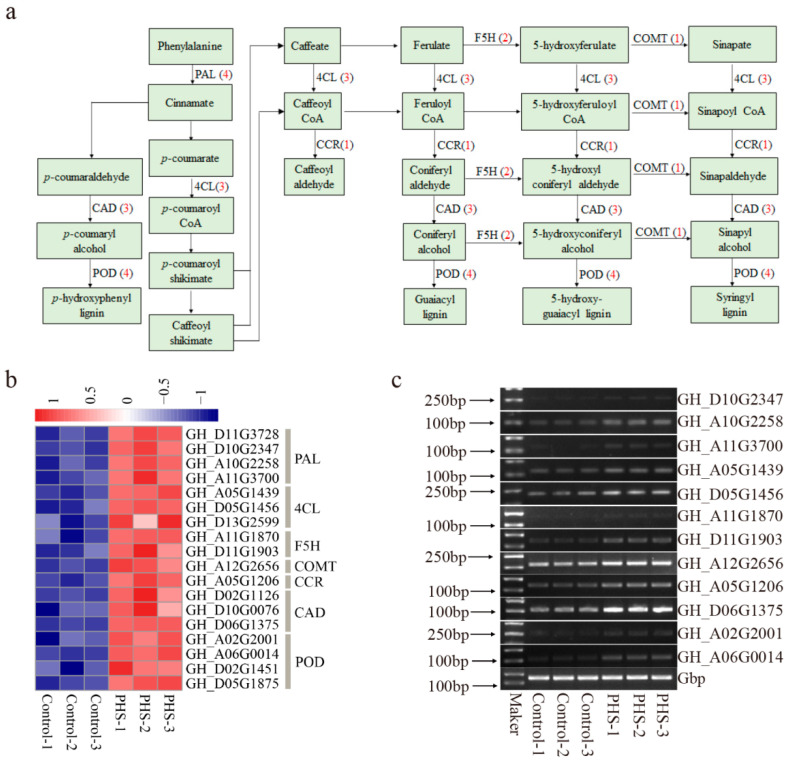
PHS induces gene expression related to phenylpropanoid biosynthesis in cotton fibers. (**a**,**b**) Up-regulated genes from the phenylpropanoid biosynthesis pathway. The numbers in red represent genes upregulated at the PHS-treated fibers. (**c**) Semi-quantitative PCR of the phenylpropanoid biosynthesis-related genes in control and PHS-treated fibers. DL2000 DNA marker (**left**). Amplified genes of interest (**right**).

The KEGG pathway analysis showed that phenylpropanoid biosynthesis pathway genes were the most enriched. A total of 18 up-regulated phenylpropanoid biosynthesis related genes were identified (Figure 6a,b), including 4 *phenylalanine ammonialyase* (*PAL*), 3 *4-coumarate-CoA ligase* (*4CL*), 1 *cinnamoyl-CoA reductase* (*CCR*), 1 *caffeic acid 3-O-methyltransferase* (*COMT*), 3 *cinnamyl alcohol dehydrogenase* (*CAD*), 2 *ferulic acid-5-hydroxylase* (*F5H*), and 4 *peroxidase* (*POD*). We randomly selected 3 *PALs*, 2 *4CLs*, 2 *F5Hs*, 1 *COMT*, 1 *CCR*, 1 *CAD,* and 2 *PODs* to verify their changes in transcript levels by a semi-quantitative PCR. Our results showed that the expression of each gene was consistent with the transcriptome data (Figure 6c).

### 2.5. Sphingosine Alters the Expression of Transcription Factor

**Figure 7:**
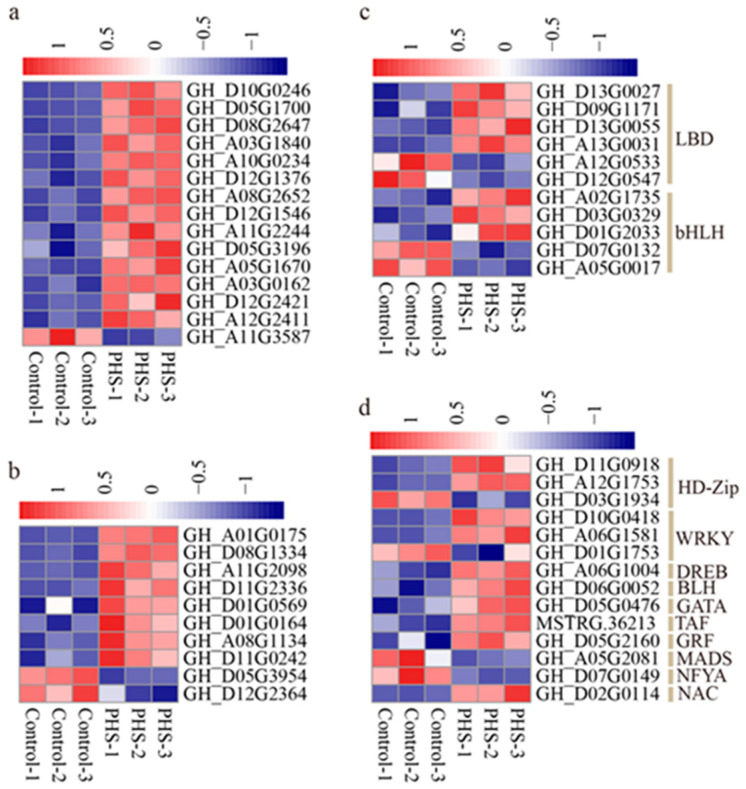
Heat map of differentially expressed transcription factors in control and PHS-treated fibers. (**a**) *MYB*. (**b**) *ERF*. (**c**) *LBD* and *bHLH*. (**d**) Other transcription factors, including *HD–Zip*, *WRKY*, *DREB*, *BLH*, *GATA*, *TAF*, *GRF*, *MADS*, *NFYA*, and *NAC*.

Numerous studies have shown that transcription factors are involved in cotton fiber development [1]. This study found that *MYBs* and *ERFs* were the transcription factor families with the largest number of genes regulated by PHS, with 15 and 10 differentially expressed genes, respectively (Figure 7a,b), and most of them were upregulated in PHS-treated fibers, while only one *MYB* and two *ERFs* were downregulated. In addition, there were 6 *LBDs*, 5 *bHLHs*, 3 *HD–Zips*, 3 *WRKYs*, 1 *DREB*, *BLH*, *GATA*, *TAF*, *GRF*, *MADS*, *NFYA,* and *NAC* expression changes. Among them, 2 *LBDs*, 2 *bHLHs*, 1 *HD–Zip*, 1 *WRKY*, 1 *MADS*, and 1 *NFYA* were downregulated, and other transcription factors were upregulated (Figure 7c,d).

### 2.6. Sphingosine Regulates Auxin-Signaling Pathway in Cotton Fiber Cells

**Figure 8:**
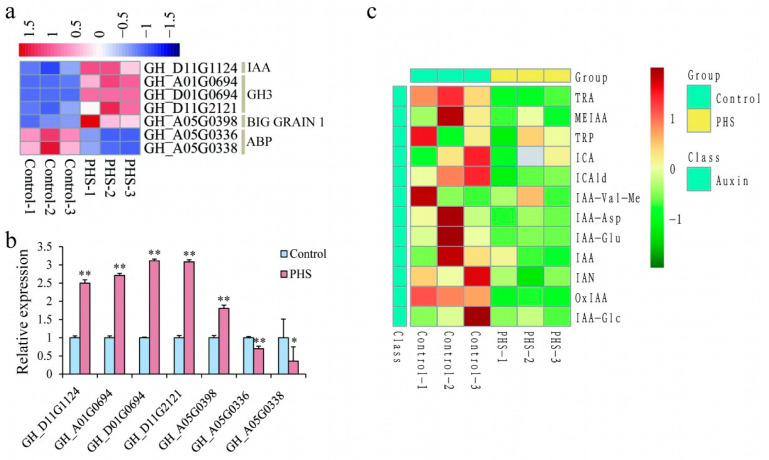
Heat map of differentially expressed genes and metabolites related to auxin in control and PHS-treated fibers. (**a**) Heat map of auxin-signaling pathway gene expression in the control and PHS-treated fibers after culturing for 10 days. (**b**) Relative expression of auxin-signaling pathway genes in the control and PHS-treated fibers after culturing for 10 days. (**c**) Heat map of auxin metabolites content in the control and PHS-treated fibers after culturing for 10 days * indicates *p* ≤ 0.05; ** indicates *p* ≤ 0.01.

Auxin regulates many aspects of plant growth and development and also plays an important role in fiber development. The expression changes in auxin-related genes were also analyzed in this study. In total, 7 auxin-signaling-pathway-related genes were affected in PHS-treated samples, including 1 *IAA*, 3 *GH3s*, 1 *BIG GRAIN 1*, and 2 *ABPs* (Figure 8a). Among these genes, *IAA*, *GH3s*, and *BIG GRAIN 1* were upregulated, while *ABPs* were downregulated (Figure 8a,b). A total of 12 auxin species were detected by LC–MS/MS. The screening criteria for differential metabolism were a fold change ≥ 2 or ≤ 0.5, and *p*-value ≤ 1; thus, TRA, IAA-Asp, and IAA-Glu were downregulated in the PHS-treated fibers, compared to the control. Nine other auxin metabolites, including free auxin (IAA), did not change between the control and PHS-treated fibers (Figure 8c). These results suggest that sphingosine can alter auxin-signaling pathways and auxin metabolism.

## 3. Discussion

Sphingolipids, as a major component of membrane lipid rafts, not only affect the structural components of biofilms but also the active molecules of various signaling pathways that affect plant growth and stress response [22,23,24,25]. A previous study found that the sphingolipid synthesis inhibitor, FB1, could lead to the disruption of sphingolipid homeostasis between cotton fibers and embryos, increasing the content of most simple sphingolipids, while reducing the content of complex sphingolipids, which seriously inhibited the elongation of cotton fibers [19]. FB1 can also inhibit cotton embryo growth, and sphingosine is main sphingolipid molecule in cotton fibers and ovules/embryos [20,21]. Previous research revealed the mechanism of sphingosine-promoting embryonic development, but the functional mechanism of sphingosine in fibers is still unclear.

This study showed that myriocin could inhibit cotton fiber elongation, while two sphingosine molecules DHS and PHS could promote early cotton fiber elongation. The application of PHS can also lead to changes in individual sphingolipid components in cotton fibers. PHS treatment can absorb more PHS (LCB t18:0) in cotton fiber, but the content of LCB t18:1 decreases and the content of LCB-1P t18:0 increases. We speculate that the increase in LCB t18:0 is not conducive to desaturation but to promote phosphorylation. Moreover, the content of Cer (t18:1/18:0, t18:1/24:0 and t18:1/28:0) and GluCer (t18:1/h18:0, t18:1/h22:0 and t18:1/h24:0) containing t18:1 decreased. The FB1 treatment results in the contents of the GluCer and GIPC of complex sphingolipids in cotton fibers and embryos were only 46.09% and 33.65% of the control, respectively, which may lead to cotton fibers elongation inhibition [19]. The GluCer in PHS-treated fibers was 80.1% of that in the control fibers, while the GIPC was not different. Moreover, PHS treatment did not increase the total sphingolipid content in fibers, while FB1 treatment resulted in 2.27 times of the total sphingolipid content in cotton fibers and embryos. The content of individual sphingolipids is affected by both PHS and FB1 treatment; FB1 also leads to the accumulation of sphingosine in cotton fibers and embryos. PHS promotes fiber elongation, while FB1 inhibits fiber elongation. Therefore, modification of the composition of sphingolipids in fibers can effectively regulate cotton fiber development, which involves a complex mechanism of sphingolipids regulation. It also provides a new angle for us to study the mechanism of cotton fiber elongation. In the future, we will explore the synthetic and metabolic regulatory genes related to sphingolipids in cotton and study their functions in cotton fiber development.

The monomer, lignin, can be formed through the phenylpropanoid biosynthesis pathway, which affects the formation and strength of the cell wall. Fibers are extremely elongated single cells, which need the close cooperation of the cell wall in the process of cell expansion. Phenylpropanoid-metabolic pathways and products are present in developing cotton fiber cells. *WLIM1a* induces expression of the *PAL-Box* gene in the phenylpropanoid biosynthesis pathway and increases phenylpropanoid-metabolic products, thus promoting fiber elongation [26]. In this study, we found that 18 phenylpropanoid-biosynthesis-related genes were upregulated by PHS, which may affect the elongation of cotton fiber by regulating the development of the cotton fiber cell wall. In future studies, we will observe the changes in fiber cell wall after PHS treatment and analyze the content changes in compounds in the phenylpropanoid biosynthesis pathway to further analyze the mechanism of PHS in regulating cotton fiber development.

Transcription factors play an important role in the development of cotton fiber cells, especially MYB transcription factors [27,28,29]. RNA interference with the expression of *GhMYB109* and *GhMYB25* leads to the shortening of fiber length [28,29]. The silencing of the *GhMYB25-like* gene produces fiber-free cotton seeds, which act upstream of *GhMYB109* and *GhMYB25* [30] (Walford et al., 2011). *GhMML3_A12* is an important gene regulating the development of cotton short fiber, which is downregulated by the self-splicing of small RNA and regulates the development of cotton fiber [31]. These studies fully demonstrated that interfering with the expression of *MYB* genes in cotton inhibited fiber development. In this study, we found that 14 *MYB* transcription factors were induced by PHS, and these *MYBs* may play an important role in PHS-regulated fiber elongation. Other transcription factors such as *HD–Zip* and *WRKY* are also involved in cotton fiber development. *GhHD1* is an HD–Zip IV family transcription factor, which increases the number of fiber initiation after overexpression and may act downstream of *GhMYB25-like* [32]. At the early stage of fiber development, GhWRKY16 directly binds to the promoters of *GhHOX3*, *GhMYB109*, *GhCesA6D–D11*, and *GhMYB25* to induce their expression, thereby promoting fiber initiation and elongation [33]. In our study, we also found that the expression of *HD–Zip* and *WRKY* was regulated by PHS. Sphingolipids, as components of lipid rafts in cell membrane, may be involved in a variety of signal transduction by affecting the activity of lipid rafts. We hypothesized that the activation and inhibition of transcription factors, especially MYB transcription factors, is an important way for sphingolipids to regulate cotton fiber development.

Auxin is an important hormone-regulating cotton fiber elongation. During the in vitro ovule culture, adding a certain concentration of auxin can promote fiber yield, while auxin antagonist PCIB can reduce fiber yield [34]. The specific promoter of cotton ovule epidermis, FBP7, drives the expression of the iaaM gene, which leads to increased auxin synthesis and accumulation in cotton ovule, promotes fiber initiation, and improves cotton yield and quality [35]. We also found that genes involved in auxin response, transport, and binding were induced by PHS. Although we detected no downregulation of free IAA content in the PHS-treated fibers or the control fibers, the content of auxin metabolites, TRA, IAA-Asp, and IAA-Glu, decreased in the PHS-treated fibers. In future studies, we need to clarify the functions of these auxin metabolites in fibers and the regulatory effect of PHS on auxin metabolism and fiber elongation.

## 4. Materials and Methods

### 4.1. Cotton Materials and In Vitro Ovule Culture

The upland cotton variety, TM-1, was planted in the experimental field of Zhengzhou, Henan Province. The cotton balls on the day of flowering were listed and picked two days later for disinfection (0.1% aqueous mercuric chloride). Embryos were stripped and placed in a Beasley and Ting’s medium [36] and treated at 32 °C for 5 and 10 days in the dark. Myriocin (0.2 µM, 1 µM, or 2 µM) with DHS (2 µM or 6 µM), or PHS (2 µM, 6 µM, or 20 µM) (Sigma, St. Louis, MO, USA) was added to the BT medium. Fibers grown for 5 or 10 days were boiled in boiling water and then rinsed with water to measure length with vernier calipers.

### 4.2. Lipid Extraction and Lipidomics

Fibers (without embryos) treated with 6 µM PHS for 10 days were used for lipidomic analysis. After sample collection, lipid extraction and lipidomics analysis were performed by Lipidall Technologies Co., Ltd., (http://www.lipidall.com/, accessed on 25 February 2021). The content of different lipid molecules between the control and PHS-treated fibers was visualized using a heatmap generated through R v3.5.1 software (https://CRAN.Rproject.org/package=pheatmap, accessed on 20 April 2022).

### 4.3. RNA-Sequencing and Bioinformatic Analysis

Total RNA was extracted from the fiber samples (without embryos) treated with 6 µM PHS and cultured for 10 days and controls with RNAprep Pure Plant Kit (Tiangen, Beijing, China) according to the product instructions. RNA-sequencings were performed by Gene Denovo Biotechnology Co., (Guangzhou, China); sequencing procedures and bioinformation analysis referenced previous research methods [21].

### 4.4. Semi-Quantitative PCR

Total RNA was extracted from the PHS-treated fibers and control samples according to the instructions of the RNA Preparation Kit (Tiangen, Beijing, China). The first-strand cDNA was synthesized via a reverse transcription kit (TAKARA, Tokyo, Japan). The semi-quantitative PCR reactions were performed using 2xTaq mix (Vazyme, Nanjing, China). The PCR reaction conditions were as follows: reaction at 95 °C for 2 min; the reaction time was 15 s at 95 °C, 20 s at 55 °C, 30 s at 72 °C, 28 cycles. Three biological replicates were performed. The specific amplification primers for selected genes and reference genes (UBQ) are listed in Table S1.

### 4.5. Auxin Content Detection

Fibers (without embryos) treated with 6 µM PHS for 10 days were used for auxin analysis. The auxin contents were detected by MetWare (http://www.metware.cn/, accessed on 10 June 2022) based on the AB Sciex QTRAP 6500 LC-MS/MS platform.

## 5. Conclusions

Our results provide important sphingolipids and biological pathways involved in cotton fiber elongation. Myriocin and PHS inhibited and promoted fiber elongation in a dose–effect manner, respectively. Exogenous PHS regulates the content of other sphingolipid molecules in cotton fibers. The RNA-Seq analysis showed that most of the genes related to phenylpropanoid biosynthesis, transcription factors (especially MYBs), and auxin response. Transport and binding pathways were upregulated in PHS-treated fibers. Auxin metabolites were decreased in PHS-treated fibers. These metabolites and differentially expressed genes may be targeted to improve cotton yield and quality in the future.

## Data Availability

Publicly available datasets were analyzed in this study. These data can be found here: https://www.ncbi.nlm.nih.gov/sra/ PRJNA1132305 (accessed on 18 July 2024).

## Supplementary Materials

The following supporting information can be downloaded at: https://www.mdpi.com/article/10.3390/plants13141993/s1, Table S1: Primer sequences for semi-quantitative PCR; Table S2: Individual sphingolipid content of fibSers treated by PHS; Table S3: RNA-seq’s library information; Table S4: The list of all DEGs in control and the PHS samples. Table S5: KEGG enrichment analysis of fibers treated by with PHS.
